# Deadenylation kinetics of mixed poly(A) tails at single-nucleotide resolution

**DOI:** 10.1038/s41594-023-01187-1

**Published:** 2024-02-19

**Authors:** Young-suk Lee, Yevgen Levdansky, Yoonseok Jung, V. Narry Kim, Eugene Valkov

**Affiliations:** 1 Center for RNA Research, Institute for Basic Science, Seoul, Republic of Korea; 2 Department of Bio and Brain Engineering, Korea Advanced Institute of Science and Technology, Daejeon, Republic of Korea; 3 RNA Biology Laboratory, Center for Cancer Research, National Cancer Institute, Frederick, MD USA; 4 School of Biological Sciences, Seoul National University, Seoul, Republic of Korea

**Keywords:** RNA, RNA decay

## Abstract

Shortening of messenger RNA poly(A) tails, or deadenylation, is a rate-limiting step in mRNA decay and is highly regulated during gene expression. The incorporation of non-adenosines in poly(A) tails, or ‘mixed tailing’, has been observed in vertebrates and viruses. Here, to quantitate the effect of mixed tails, we mathematically modeled deadenylation reactions at single-nucleotide resolution using an in vitro deadenylation system reconstituted with the complete human CCR4–NOT complex. Applying this model, we assessed the disrupting impact of single guanosine, uridine or cytosine to be equivalent to approximately 6, 8 or 11 adenosines, respectively. CCR4–NOT stalls at the 0, −1 and −2 positions relative to the non-adenosine residue. CAF1 and CCR4 enzyme subunits commonly prefer adenosine but exhibit distinct sequence selectivities and stalling positions. Our study provides an analytical framework to monitor deadenylation and reveals the molecular basis of tail sequence-dependent regulation of mRNA stability.

## Abstract (web summary)

Here, using mathematical modeling and an in vitro deadenylation system, the authors quantitatively demonstrate the effect of non-adenosines in the poly(A) tail and exemplify how tail sequence regulates mRNA stability.

## Main

It has long been considered that poly(A) tails consist purely of adenosine stretches. However, the development of methods such as 3′-untranslated region and poly(A) tail region sequencing (TAIL-seq) enabled the sequencing of poly(A) tails and revealed that some messenger RNA poly(A) tails contain intermittent non-adenosine (non-A) residues^1,2^. Recent studies based on long-read sequencing also demonstrated the widespread presence of mixed tails^3,4^. Such ‘mixed’ poly(A) tails are a consequence of the enzymatic activity of terminal nucleotidyltransferases TENT4A (also known as PAPD7, TRF4, TUT5 and POLS) and TENT4B (also known as PAPD5, GLD4, TRF4-2 and TUT3)^5,6^. While the TENT4 homologs do favor adenosine and thus were initially considered to be poly(A) polymerases, they can incorporate non-A residues, albeit less efficiently than adenosines^2,7^. Among non-As, guanosine is preferred, followed by cytosine and uridine.

In vertebrates, TENT4 assembles into two types of complexes: the nuclear TRAMP complex composed of ZCCHC7, MTR4 and TENT4 (mainly TENT4B)^8^ and the cytosolic complex, which consists of ZCCHC14 and TENT4^9,10^. The TRAMP complex modifies various nucleoplasmic/nucleolar transcripts to facilitate their maturation or decay by 3′-to-5′ exoribonucleases^8^. In contrast, the cytosolic TENT4–ZCCHC14 complex acts on mRNAs to extend their poly(A) tails, delaying the deadenylation process and increasing the mRNA half-life^10^.

Mixed tailing is observed on mRNAs of at least one-fifth of genes in vertebrates^1,2^. Some viruses co-opt the mixed tailing machinery to promote their proliferation^10^. For example, transcripts of hepatitis B virus and human cytomegalovirus (HCMV) contain specialized *cis*-acting elements with a CNGGN pentaloop, namely the post-transcriptional regulatory element^11^ and SL2.7^10^, respectively. These elements recruit ZCCHC14, which brings TENT4 to the viral RNAs, resulting in mixed tailing and stabilization of viral transcripts. The post-transcriptional regulatory element of the woodchuck hepatitis virus, which harbors two CNGGN pentaloops, is widely used to enhance transgene expression from plasmids and viral vectors^12,13^.

Shortening of the poly(A) tail is a rate-limiting step in cytoplasmic mRNA decay^14,15^. The multisubunit CCR4–NOT complex is the principal factor regulating the length of the poly(A) tails of most eukaryotic transcripts^16,17^. It possesses two catalytic subunits: a CCR4 homolog belonging to the endonuclease/exonuclease/phosphatase-type exonuclease family and a CAF1 homolog, a DEDD-type exonuclease^18,19^. Humans have two CCR4 paralogs (CCR4a/CNOT6 and CCR4b/CNOT6L) and two CAF1 paralogs (CAF1/CNOT7 and POP2/CNOT8/CALIF). Each catalytic subunit has a distinct function: CCR4 trims poly(A) tails coated with cytoplasmic poly(A) binding protein, while CAF1 is active on poly(A)-free of poly(A) binding protein^17,19^. In addition, the mammalian CCR4–NOT complex contains six non-enzymatic subunits: CNOT1, CNOT2, CNOT3, CNOT9/CAF40, CNOT10 and CNOT11^20^. CNOT1 serves as the essential scaffold on which the complex assembles^21^. CNOT9, CNOT2 and CNOT3 physically interact with RNA-binding proteins to elicit transcript-specific deadenylation^22^ and decapping^23^. The reconstitution of the complete human CCR4–NOT complex from purified recombinant components revealed at least two of the three nonenzymatic modules (CNOT9, CNOT10:CNOT11 and CNOT2:CNOT3) are required for maximal deadenylation activity^20^.

Increasing experimental evidence indicates that the non-A residues within the mixed tail negatively impact deadenylation and extend the mRNA half-life, that is, in addition to the adenylation activity, which extends the length of a poly(A) tail^2,10,24,25^. To assess the quantitative impact of mixed tailing on deadenylation, it became necessary to establish a mathematical framework and rigorously validate the derived kinetic parameters with biochemical data. Two critical experimental considerations for the successful implementation of mathematical modeling were (1) the ability to assay deadenylation as a time course with single-nucleotide resolution and (2) strict compositional control in a fully recombinant system with the ability to incorporate catalytic mutations in individual subunits. In this Article, we describe in vitro deadenylation assays and estimate the deadenylation kinetics on pure poly(A) and mixed-tailed substrates in precisely controlled biochemical contexts. This approach offers a unique opportunity to measure the exact impact of mixed tailing in the context of deadenylation.

## Results

### Dynamic model of deadenylation kinetics

**Extended Data Fig. 1:**
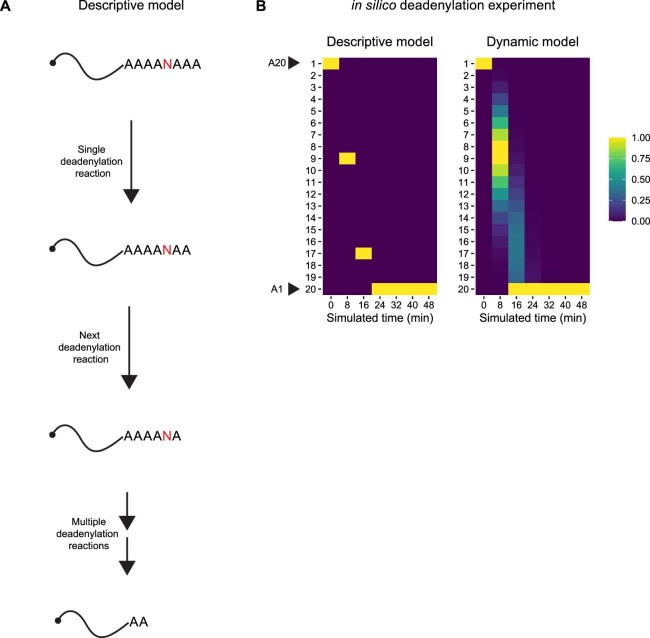
Mathematical models of deadenylation. **a**. Schematic of a descriptive model of deadenylation. **b**. *In silico* deadenylation experiment and heatmap analysis comparing the descriptive and dynamic models. Column-specific unity-based normalization was applied for data visualization. A20 represents RNA with a poly(A) tail length 20, and A1 represents RNA with a single-adenosine tail.

To measure the deadenylation kinetics of mixed tails, we designed a mathematical model that does not assume a constant reaction rate for each nucleotide but instead accounts for the possible changes in kinetics within a molecule, for example, when encountering a non-adenosine residue. Existing methods estimate the average reaction rate by computing the modal poly(A) tail length and then fitting this to a linear model^26^. This approach inadvertently assumes a descriptive model in which all the molecules in the reaction undergo deadenylation at the same time (Extended Data Fig. 1a). Previous analyses of biochemical deadenylation experiments^26^ were based on the concept of a ‘modal’ poly(A) tail length: that is, the poly(A) tail length of a single most abundant RNA species observed at a given time point. This approach is mathematically equivalent to a descriptive model and does not take into account the deadenylation dynamics of other RNA species with different, ‘non-modal’ poly(A) tail lengths.

**Fig. 1:**
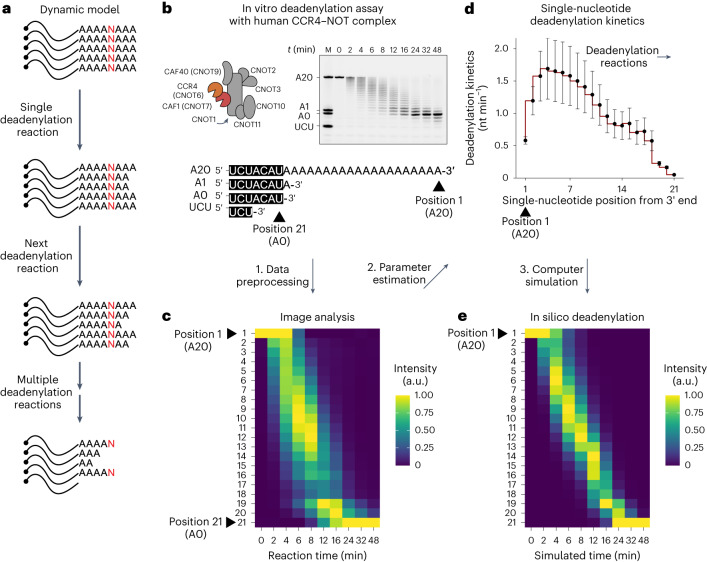
Mathematical modeling of deadenylation kinetics at single-nucleotide resolution. **a**, Schematic of a dynamic model of deadenylation. **b**, In vitro deadenylation assay with human CCR4–NOT complex. **c**, Heatmap presentation of the deadenylation assay data. Position 1 represents the A20 RNA substrate, and position 21 represents the tailless 7-mer RNA. Column-specific unity-based normalization was applied for data visualization. **d**, Parameter estimation and deadenylation kinetics at each single-nucleotide position from the 3′ end. Error bars represent the standard error of parameter estimation. **e**, In silico deadenylation based on the estimated parameters. Column-specific unity-based normalization was applied for data visualization.

To address this, we decided to develop an analytical framework in which biochemical reactions are dynamic rather than deterministic events, which is an intrinsic property of in vitro deadenylation experiments (Fig. 1a). We further assume that the deadenylation process follows the first-order Markov property to mathematically decouple the kinetics of hydrolysis of each nucleotide (see Methods for details). For example, under the first-order Markov property, the hydrolysis rate of the second adenosine is independent of that of the first adenosine. This model resembles previous mathematical models of deadenylation^15,27^ but does not assume constant kinetics. Instead, our approach is analogous to the mathematical model used to measure the polyadenylation kinetics of the TRAMP complex on transfer RNA^28^. Simulated deadenylation using the dynamic model exhibits a distribution of multiple deadenylation intermediates, unlike the descriptive model that gives a single intermediate at a given time point (Extended Data Fig. 1b). This observation of an improved fit to biochemical data confirms that our model more accurately recapitulates the dynamics of deadenylation.

We then tested our model on an in vitro deadenylation experiment with the complete human CCR4–NOT complex (Fig. 1b). A raw gel image with deadenylation products and intermediates was pre-processed to measure the amount of each RNA intermediate (Fig. 1c) (see Methods for details). The parameters of our model corresponded to the deadenylation kinetics at each nucleotide and were estimated using the Levenberg–Marquardt (LM) algorithm^29^, a general algorithm for estimating parameters of a nonlinear model (Fig. 1d). Computer simulation based on these estimated parameters generates a distribution of RNA species similar to that of the in vitro deadenylation experiment (Fig. 1e), indicating that the parameters of our dynamic model are reliable estimates of single-nucleotide deadenylation kinetics.

### CCR4–NOT stalls at multiple positions relative to guanosine

**Fig. 2:**
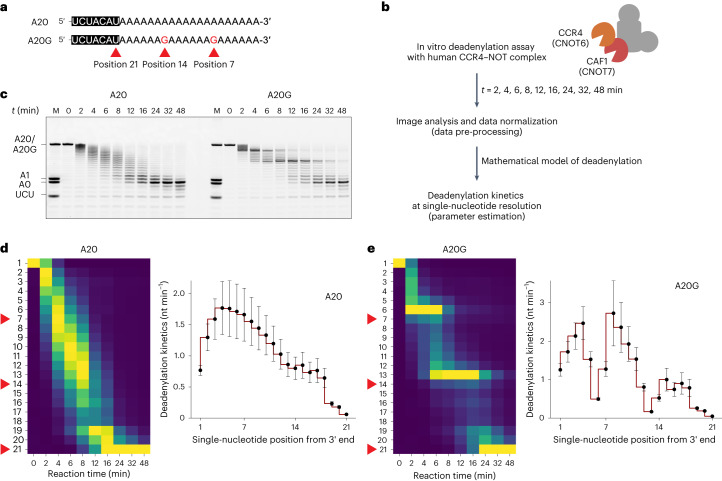
Stalling effect of a single guanosine residue on human CCR4–NOT complex. **a**, Sequences of the synthetic 7-mer-A20 and 7-mer-A20G RNA substrates. Note the two guanosine residues at positions 7 and 14 for A20G. Position 21 represents the tailless 7-mer RNA. **b**, Schematic of the in vitro deadenylation analysis with human CCR4–NOT complex. **c**, In vitro deadenylation experiment with A20 and A20G RNA substrates (50 nM) and wild-type CCR4–NOT complex (25 nM). **d**, Left: heatmap analysis for A20 substrates. Column-specific unity-based normalization was applied for data visualization. Red arrowheads indicate the single-nucleotide positions 7, 14 and 21 from the 3′ end. Right: estimated deadenylation kinetics (nt min^−1^) based on the dynamic model. Error bars represent the standard error of parameter estimation. **e**, Deadenylation analysis as in **d** but with A20G RNA substrates. Error bars represent the standard error of parameter estimation. Heatmaps in **d** and **e** follow the same colour scale as in Fig. 1c,e. Source data

To assess the impact of mixed tailing during CCR4–NOT-mediated deadenylation, we designed synthetic RNA substrates with pure poly(A) tail sequences or mixed tails (Fig. 2a). The substrate with a pure poly(A) tail (A20) contains a ‘body’ composed of seven nucleotides (5′-UCUACAU-3′) followed by a homopolymeric poly(A) stretch of 20 nt. The mixed-tailed substrate (A20G) is identical to A20 except for the two guanosine residues at positions 7 and 14 from the 3′ end. Of note, in our previous work, we utilized synthetic RNAs with a terminal or penultimate guanosine to measure the reaction rate of 3′-to-5′ trimming^2^. But this earlier work did not take into account the possibility of the random incorporation of non-A residues within the poly(A) tail. By embedding the guanosine well within the poly(A) tail, these substrates better reflect the physiological scenarios and permit a comprehensive survey of the substrate specificity of deadenylases.

The in vitro deadenylation experiment was conducted with the human CCR4–NOT complex consisting of all eight core subunits, including CCR4a/CNOT6 and CAF1/CNOT7^20^. The products were resolved by denaturing polyacrylamide gel electrophoresis^26^. In particular, the experiments were conducted for multiple reaction time points (that is, 2, 4, 6, 8, 12, 16, 24, 32 and 48 min) to achieve high-resolution measurements of the change in RNA abundance at each nucleotide position (Fig. 2b). Subsequent image analysis, data pre-processing and parameter estimation was applied to measure the deadenylation kinetics at single-nucleotide resolution (nucleotides per minute; nt min^−1^).

As expected, with the A20 substrate, we observed no RNA accumulation near positions 7 and 14 (Fig. 2c, left and Fig. 2d, left). Substantial RNA accumulation started at position 19 and afterward, suggesting that stalling of the deadenylation process begins at the antepenultimate (or −2) position relative to the seven-nucleotide body. It is worth emphasizing that the deadenylation estimates are maximum likelihood estimates, and the error bars represent the range of the true parameter value (see Online Methods for details). Unexpectedly, the deadenylation kinetics was not constant as a function of the poly(A) tail. Instead, we observed an increase in the deadenylation rate for the first four nucleotides followed by a gradual deceleration (Fig. 2d, right). The relatively low rate at the beginning may reflect the lag time for the complete assembly of the enzyme–substrate complex. The subsequent decrease may be due to the low processivity of the deadenylases, which stochastically dissociate from the substrate.

**Extended Data Fig. 2:**
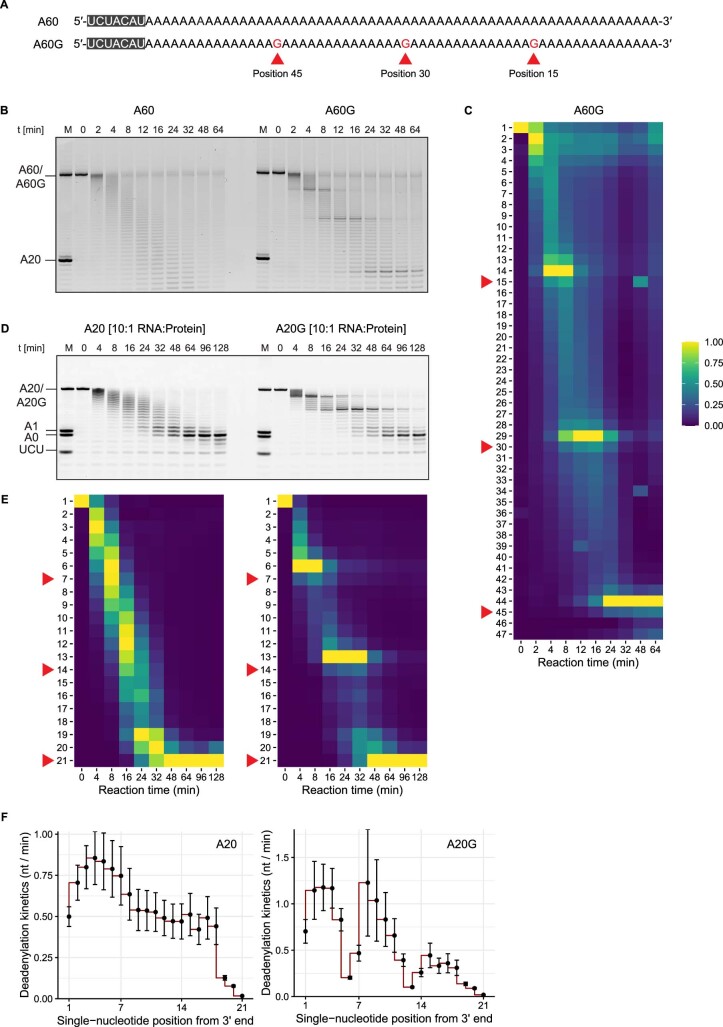
Alternative conditions for *in vitro* deadenylation experiments. **a**. Sequences of the synthetic 7-mer-A60 and 7-mer-A60G RNA substrates. **b**. Representative raw gel images from *in vitro* deadenylation experiment with A60 and A60G RNA substrates (50 nM) and wildtype CCR4-NOT complex (25 nM) of at least three technical and two biological replicates. **c**. Heatmap analysis for A60G substrates in (B). Column-specific unity-based normalization was applied for data visualization. Red arrowheads indicate the single-nucleotide positions 15, 30, and 45 from the 3′ end. **d**. Representative raw gel images after five-fold dilution from *in vitro* deadenylation experiment with A20 and A20G RNA substrates (250 nM) and wildtype CCR4-NOT complex (25 nM) of at least three technical and two biological replicates. **e**. Heatmap analysis for A20 and A20G substrates in (D). Column-specific unity-based normalization was applied for data visualization. Red arrowheads indicate the single-nucleotide positions 7, 14, and 21 from the 3′ end. **f**. Estimated deadenylation kinetics (nt / min) based on the dynamic model of (E). Error bars represent the standard error of parameter estimation. Source data

For the A20G substrates, we observed a substantial accumulation at positions 6 and 13 (Fig. 2c, right and Fig. 2e, left), which are the penultimate (or −1) positions to the respective guanosine residues. We then applied our mathematical model and discovered a substantial decrease in deadenylation rate at three positions (Fig. 2e, right). Pausing at the penultimate and terminal positions is consistent with our previous observations^2^. We further observe a modest but substantial stalling at the antepenultimate (−2) position. Stalling effect of guanosine is most pronounced at the −1 position (position 6), which is 2.42 times greater than at the 0 position (position 7). Similar results were observed with longer poly(A) tails of 60 nucleotides (Extended Data Fig. 2a–c) and under conditions of ten-fold substrate excess (Extended Data Fig. 2d,e), suggesting that our biochemical conditions faithfully reflect the intrinsic kinetic properties of the human CCR4–NOT complex.

### Pyrimidines are most effective in stalling deadenylation

**Fig. 3:**
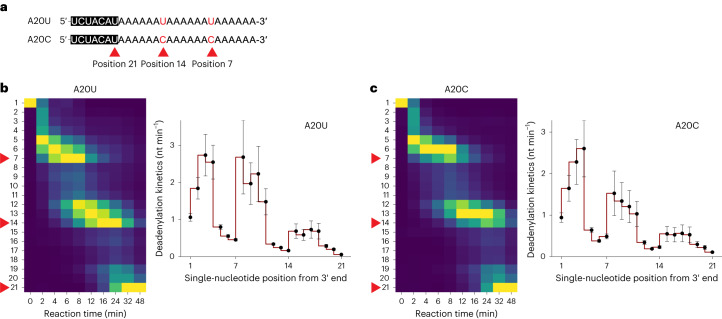
Single pyrimidine residue produces a similar stalling effect but with greater impact at the terminal and antepenultimate positions. **a**, Sequences of the synthetic 7-mer-A20U and 7-mer-A20C RNA substrates (50 nM) and wild-type CCR4–NOT complex (25 nM). Note the two pyrimidine residues at position 7 and 14 for A20U and A20C. Position 21 represents the tailless 7-mer RNA. **b**, Left: heatmap analysis for A20U substrates. Column-specific unity-based normalization was applied for data visualization. Red arrowheads indicate the single-nucleotide positions 7, 14 and 21 from the 3′ end. Right: estimated deadenylation kinetics (nt min^−1^) based on the dynamic model. Error bars represent the standard error of parameter estimation. **c**, Deadenylation analysis as in **b** but with A20C RNA substrates. Error bars represent the standard error of parameter estimation. Heatmaps in **b** and **c** follow the same colour scale as in Fig. 1c,e.

To investigate the impact of other non-A residues, we designed synthetic RNA substrates with two intermittent uridine or cytidine residues instead of guanosine (Fig. 3a). TENT4 enzymes incorporate not only adenosines and guanosines but also uridines and cytidines, albeit at lower frequencies^2^. In the context of targeted mixed tailing, such as in HCMV RNA2.7, at least 10% of their 3′ end tails contain single pyrimidine residues^10^. This suggests that pyrimidines may contribute substantially to the overall decrease of the deadenylation rate although the contribution of pyrimidines has been largely overlooked. Stalling of uridine and cytidine residues was observed with both human CAF1 and CCR4 proteins^2^, but the magnitude of their stalling effects remains unknown.

**Extended Data Fig. 3:**
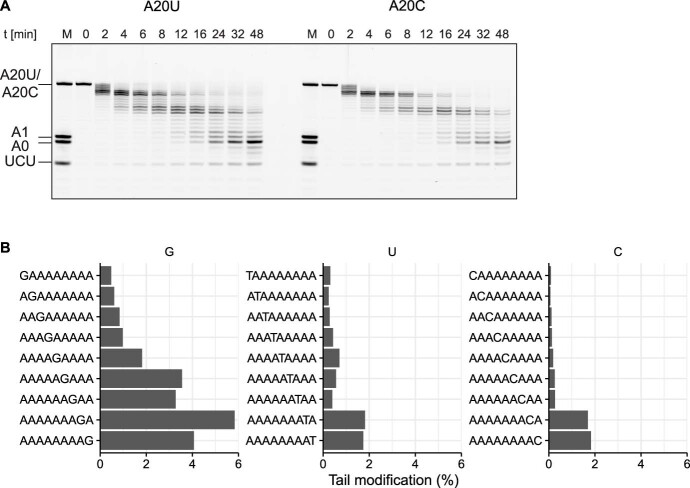
Supporting evidence related to Fig. 3. **a**. Representative raw gel images from in vitro deadenylation experiment with A20U and A20C RNA substrates (50 nM) and wildtype CCR4-NOT complex (25 nM) of at least three technical and two biological replicates. These images were used to quantitate deadenylation kinetics shown in Fig. 3. **b**. Re-analysis of TAIL-Seq data on HCMV-infected cells 10. The fraction of modified tails of HCMV RNA2.7 was calculated for RNAs with a poly(A) tail length of ≥25 nt (n = 1 TAIL-seq experiments). Source data

With the pyrimidine-containing substrates (A20U and A20C), we observed RNA accumulation at three positions: antepenultimate (−2), penultimate (−1) and terminal (0) positions (that is, positions 5, 6, 7 and 12, 13, 14) (Fig. 3b, left, Fig. 3c, left and Extended Data Fig. 3a). Modeling revealed that the deadenylation rates at −2 positions were comparable to their respective −1 and 0 positions (Fig. 3b, right and Fig. 3c, right). Compared to our analysis of guanosine residues, the removal rates of uridine and cytidine are lower, particularly at the −2 and 0 positions, which is evident by the distinct accumulation pattern of the pyrimidine experiments. Therefore, single pyrimidine residues exhibit a greater stalling effect than guanosine residues owing to the position-dependent specificity of the human CCR4–NOT complex. Moreover, this indicates that the CCR4–NOT slows down already two nucleotides in advance of encountering any non-A residue, hinting at the possibility that its molecular basis of poly(A) recognition lies on the three nucleotides of the 3′ end.

To investigate the physiological relevance of this accumulation at the −1 position, we re-examined our TAIL-seq data on HCMV-infected cells^10^. Previously, we found that HCMV RNA2.7 is highly expressed and undergoes extensive mixed tailing. Tail modification at the 0 and −1 positions of HCMV RNA2.7 is considerably higher than other positions for all three non-As (Extended Data Fig. 3b), which is consistent with paused deadenylation induced by non-A residues in vitro. Of note, tail modification at the −1 position has been largely overlooked owing to the abundance of this modification being generally low in mammalian cells^1,2^. We further found that tail modification at the −2 position is less prominent in cells than in vitro, which suggests that other cellular *trans*-factors may prevent the accumulation of modified tails at the −2 position.

### Stalling behavior of CAF1

**Fig. 4:**
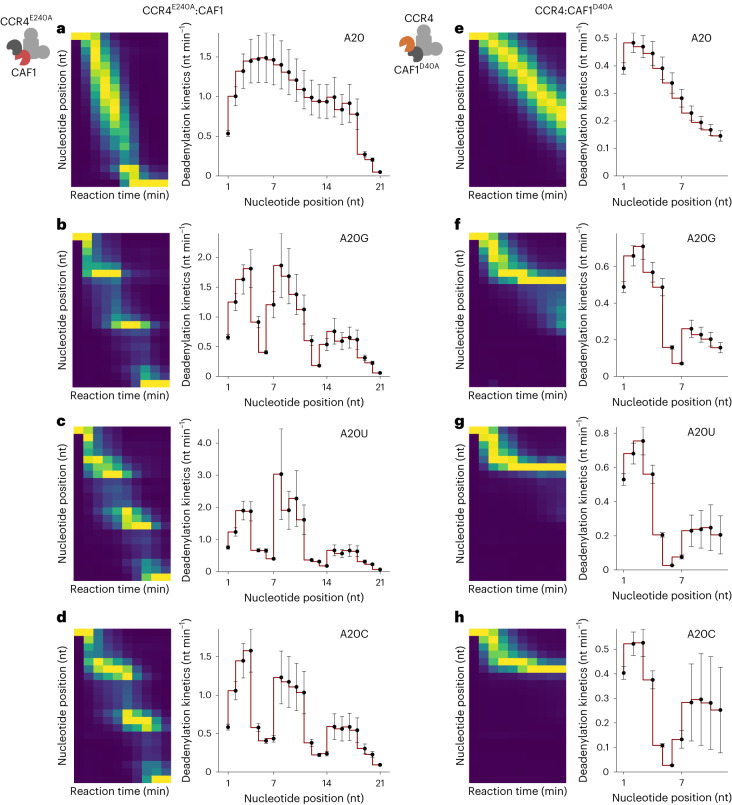
The distinct kinetic property of each catalytic subunit. **a**–**d**, CCR4^E240A^:CAF1 mutant deadenylation analysis with A20 (**a**), A20G (**b**), A20U (**c**) and A20C (**d**) RNA substrates. **e**–**h**, CCR4:CAF1^D40A^ mutant deadenylation analysis with A20 (**e**), A20G (**f**), A20U (**g**) and A20C (**h**) RNA substrates. Left: heatmap analysis. Column-specific unity-based normalization was applied for data visualization. Incubation was done for up to 48 min as in Fig. 3. Right: estimated deadenylation kinetics (nt min^−1^) based on the dynamic model. Error bars represent the standard error of parameter estimation. Heatmaps in **a**–**h** follow the same colour scale as in Fig. 1c,e.

**Extended Data Fig. 4:**
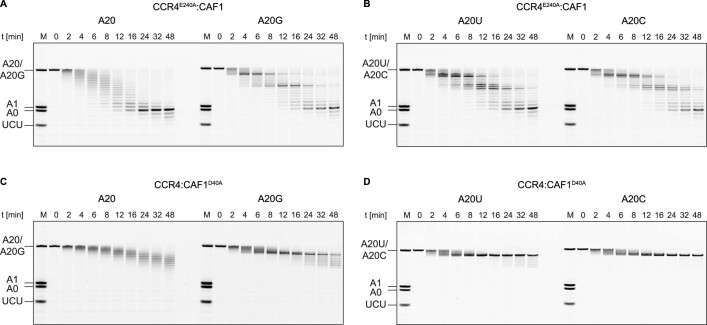
Supporting evidence related to Fig. 4. Representative raw gel images from *in vitro* deadenylation experiments with catalytic mutants (A,B) CCR4^E240A^:CAF1 and (C,D) CCR4:CAF1^D40A^ of at least three technical and two biological replicates. These images were used to quantitate deadenylation kinetics shown in Fig. 4. Source data

The CCR4–NOT complex contains two distinct catalytic subunits: CCR4 (CNOT6 or CNOT6L) and CAF1 (CNOT7 or CNOT8). Previously, we reported that CAF1 stalls at the penultimate (−1) while CCR4 stalls at the 3′ terminus (0) of guanosine residues^2^, suggesting that the two enzymes exhibit differential selectivity for non-A residues in poly(A) tails. We sought to examine the contribution of each enzyme in the context of the entire CCR4–NOT. First, we conducted in vitro deadenylation experiments using the CCR4–NOT complex reconstituted with wild-type CAF1 and a catalytic mutant of CCR4 (E240A)^30^, thus ensuring that CAF1 is the only active deadenylase subunit. Structural predictions and modeling of the *Schizosaccharomyces* *pombe* Caf1 protein interacting with a polyadenosine sequence suggested that a helical structure resulting from base-stacking effects in polyadenosine is recognized by the active site of the CAF1 enzyme, which can accommodate up to five nucleotides^24^. No noticeable kinetic changes were observed near positions 7 and 14 of the A20 substrate (Fig. 4a and Extended Data Fig. 4a) as with the wild-type complex. However, with the mixed-tailed A20G, deadenylation rates substantial decreased at positions 5 and 6 and positions 12 and 13 (Fig. 4b and Extended Data Fig. 4a), which are the antepenultimate (−2) and penultimate (−1) positions relative to the guanosine residues. The stalling effect at the terminal (0) positions was less pronounced in comparison.

With uridine and cytidine substitutions, a substantial decrease in the deadenylation rates occurred at all three nucleotide positions (Fig. 4c,d and Extended Data Fig. 4b), consistent with CAF1 recognizing the pyrimidine residue two nucleotides in advance. This suggests that CAF1’s substrate specificity lies in the last three nucleotides of the poly(A) tail.

### CCR4 is highly specialized for pure poly(A) tails

The second catalytic subunit of the CCR4–NOT complex is CCR4 (CNOT6 or CNOT6L). It is tethered to the CNOT1 scaffold protein via CAF1^31^, and it belongs to the endonuclease/exonuclease/phosphatase exonuclease family^32^. The structure of the catalytic domain of human CCR4 and poly(A) DNA revealed a possible three-nucleotide pocket that may be responsible for its poly(A) specificity^30^.

To investigate CCR4’s contribution to deadenylation, we reconstituted the CCR4–NOT complex with a catalytic mutant of CAF1(D40A)^33^, leaving CCR4 as the only active deadenylase subunit in the complex. Note that, for these CAF1 mutant experiments, the deadenylation process did not complete within 48 min (Fig. 4e and Extended Data Fig. 4c). To preserve the integrity of the modeling, we only estimated the first 11 positions from the 3′ end, including the first non-A residue at position 7 (see Methods for details).

A20G exhibited substantial accumulation at position 7 (Fig. 4f, left and Extended Data Fig. 4c), indicating the decrease in CCR4 activity during the hydrolysis of the 3′ terminal guanosine. Subsequent analysis revealed a substantial reduction in deadenylation rate at positions 6 and 7 (penultimate and terminal, respectively) but not at position 5 (antepenultimate) (Fig. 4f, right). This analysis indicates that CCR4 stalls at the penultimate and terminal positions but not the antepenultimate position. Based on these observations, CAF1 is primarily responsible for the kinetic slowdown at the antepenultimate (−2) position from the single guanosine residue.

The pyrimidine experiments with A20U and A20C showed a similar pattern, but principal accumulation occurred at position 6 instead of position 7 (Fig. 4g,h and Extended Data Fig. 4d), suggesting the deceleration for pyrimidine residues occurs one nucleotide earlier than for guanosine residues. Modeling revealed that the deadenylation rates decreased at all three positions but were less pronounced at the antepenultimate (−2) position. This is consistent with the notion that similar to CAF1, CCR4 also recognizes the last three nucleotides of the poly(A). Thus, CAF1 and CCR4 may exhibit similar specificity for single pyrimidine residues. However, in terms of the extent of stalling effect, CCR4 appears to be more specialized for pure poly(A) tails than CAF1.

### Quantifying the stalling effect of non-adenosine residues

**Fig. 5:**
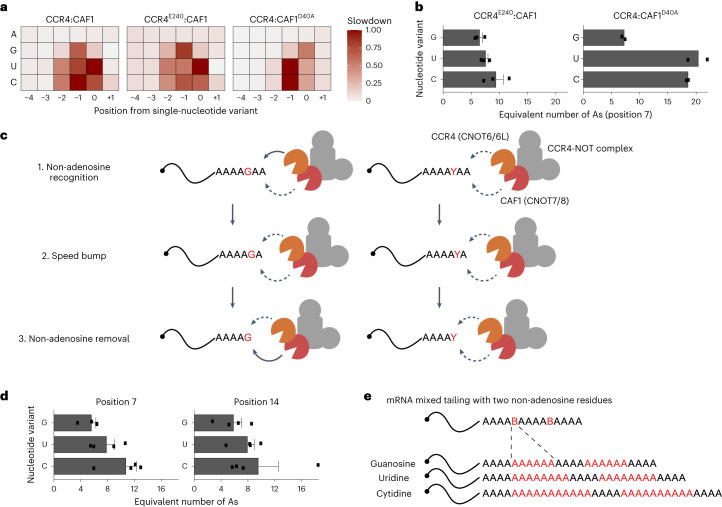
Quantitative impact of mixed tails in terms of stalling deadenylation. **a**, The relative slowdown (or time for single-nucleotide removal) for each nucleotide variant and nucleotide position from the first non-A residue (that is, position 7). Unity-based normalization was applied for each type of human CCR4–NOT complex experiment. A, adenosine; G, guanosine; U, uridine; C, cytidine. **b**, The equivalent number of additional adenosines in terms of the deadenylation activity of CCR4^E240A^:CAF1 (*n* = 3) and CCR4:CAF1^D40A^ (*n* = 2). The impact of the first residue (that is, position 7) was estimated. The barplot represents the mean number of additional adenosines, and error bars represent the s.e.m. **c**, A quantitative model of CCR4–NOT-dependent deadenylation. The solid lines represent strong deadenylation activity, and dashed lines represent relatively weak activity. **d**, The equivalent number of additional adenosines in terms of the deadenylation activity of wild-type complexes (*n* = 4). Impacts of both residues (positions 7 and 14) were estimated and are presented separately. The barplot represents the mean number of additional adenosines, and error bars represent the s.e.m. **e**, The quantitative impact of mixed tails in terms of the number of additional adenosines.

By calculating the inverse of the deadenylation rate, one can estimate the time required for nucleotide removal for that position. In effect, this removal time provides a quantitative assessment of the deadenylation specificity of the CCR4–NOT complex (Fig. 5a). The wild-type complex stalls at three positions with a guanosine residue, mainly at the −1 position. CAF1 is responsible for the stalling at the −2 position, while CCR4 pauses at 0 position relative to guanosine. With uridine or cytidine, the wild-type complex stalls at comparable levels across all three positions, −2, −1 and 0. CCR4 is especially sensitive to inhibition at the −1 position. It is worth mentioning that our analysis also suggests a modest difference between the two pyrimidines in terms of CAF1. Cytidine residues exhibit a slightly more inhibitory effect at the −1 position than uridine. At the 0 position, uridine mainly disrupts the activity of CAF1. All in all, the summed activities of CAF1 (Fig. 5a, middle) and CCR4 (Fig. 5a, right) seem to be reflected in the wild-type complex activity (Fig. 5a, left).

More importantly, this analytical framework provides the means to quantitate the equivalence of a single non-A residue to the number of adenosines in terms of the deadenylation reaction time (see Methods for details). Based on replicate experiments of independently purified CCR4–NOT complexes, we calculated the time required for the removal of a single non-A residue relative to that of adenosine (Fig. 5b). CAF1 takes 6.5 ± 0.3 times longer to remove single guanosine compared to an adenosine residue, while uridine and cytosine residues are equivalent to 7.5 ± 0.3 and 9.4 ± 0.8 adenosines, respectively (Fig. 5b, left). CCR4 is strongly inhibited by a single pyrimidine, which is equivalent to 18.4–21.6 adenosines, further highlighting CCR4’s specificity for pure poly(A). Altogether, these observations point to a mechanism of CCR4–NOT-dependent deadenylation, where each catalytic subunit reacts distinctly and selectively when it encounters non-A residues (Fig. 5c).

Finally, we applied this approach to experiments with wild-type enzymes to measure the deadenylation rate of the full CCR4–NOT complex (Fig. 5d). For the first residue (position 7), single guanosine corresponds to 5.6 ± 0.7 adenosines, uridine corresponds to 7.8 ± 1.2 adenosines and cytidine 10.7 ± 1.6 adenosines. We observed a similar trend for the second residue (position 14). However, this combined effect is less than the sum of each subunit, suggesting that the two enzymes do not simply work additively in deadenylation. This quantitative analysis reveals the exact impact of non-A residues (Fig. 5e) and an unexpected selectivity of non-A residues in the deadenylation process of the CCR4–NOT complex.

## Discussion

The discovery of non-A residues within the poly(A) tail has opened the possibility that the poly(A) tail may regulate the kinetics of deadenylation in a sequence-dependent manner^2^. Previous in vitro experiments from our laboratory and others have demonstrated this possibility but were unsuccessful in quantifying the precise stalling effect of each non-A residue^2,24,25^. To address this challenge, we designed a mathematical model that closely parameterizes the deadenylation process. With this model, we were able to describe the kinetics of deadenylation at single-nucleotide resolution. When applied to enzymatic reactions with pure poly(A) sequences, this model indicates that the rate of their hydrolysis is not constant but is in fact variable.

Further, applying the model in reactions with mixed tails revealed that the human CCR4–NOT complex stalls at the antepenultimate, penultimate and terminal positions (−2, −1 or 0) relative to a single non-A residue. Experiments with catalytically inactivated mutants hint at the distinct but dynamic roles of the two catalytic subunits of the CCR4–NOT complex (Fig. 5c). CAF1 stalls at the antepenultimate position by recognizing the non-A residue which is located 2 nt ahead. It was proposed that the active site of CAF1 is capable of accommodating as many as five nucleotides^24^. However, modification at this antepenultimate position seems not to be the dominant form based on our published TAIL-seq data. One can speculate a mechanism involving different exonucleases (for example, PAN2/3 or the exosome) or factors regulating the activity of CCR4–NOT.

While CAF1 pauses at the antepenultimate position, CCR4 removes the single adenosine to proceed with the poly(A) tail shortening process. Then, the penultimate non-A acts as a sort of ‘speed bump’ for both enzymes. This speed bump has been previously left unnoticed for it being relatively low in cells. In this study, the tail modification at the −1 position is closely re-examined as we find that it is the major single-nucleotide modification of highly mixed-tailed RNAs. It may be worth investigating beyond the steady-state tail modifications in cells and measuring its pre-steady state as done with the length of poly(A) tails^15,34^. Finally, at the terminal position, CAF1 is responsible for the removal of the single non-A residue as CCR4 instead pauses at this position. Thus, the two enzymes may take turns or may be ‘tag-teamed’ during the course of mixed tail removal. We term this the ‘tag-team’ mechanism.

To investigate the physiological relevance of this accumulation at the −1 position, we re-examined our TAIL-seq data on HCMV-infected cells^10^. Previously, we found that HCMV RNA2.7 is highly expressed and undergoes extensive mixed tailing. Tail modification at the 0 and −1 positions of HCMV RNA2.7 is considerably higher than other positions for all three non-As (Extended Data Fig. 3b), which is consistent with paused deadenylation induced by non-A residues in vitro. Of note, tail modification at the −1 position has been largely overlooked owing to the abundance of this modification being generally low in mammalian cells 1, 2. We further found that tail modification at the −2 position is less prominent in cells than in vitro, which suggests that other cellular *trans*-factors may prevent the accumulation of modified tails at the −2 position.

It is possible that this tag-team and speed bump effect of single non-As compels the CCR4–NOT complex to switch from a processive reaction to a more on–off, distributive deadenylation. That is, the intrinsic substrate specificity hints at the possibility that the processivity of these deadenylases may be interrupted by the encounter with the non-A residues. This encounter of non-A residues within the poly(A) tail may hinder the processive reaction and influence the CCR4–NOT complex to revert to distributive deadenylation. In effect, these non-A residues may act as another facet for regulating the length of the poly(A) tail. While our current in vitro conditions are not optimized for processive deadenylation, further explorations of these two modes of deadenylation with our approach may reveal additional kinetic properties of non-A residues.

Combining the effect at all three positions, we were able to quantify the equivalent number of As for a single non-A residue in the context of deadenylation. A guanosine residue is equivalent to approximately six adenosines with both enzymes, and uridine/cytidine corresponds to 8–11 adenosines (Fig. 5d,e). It is currently unknown to what extent non-As are incorporated into mRNA tails. Our earlier measurements using in vitro assays showed that mixed tails contain 20–25% of non-As when equimolar concentrations of nucleoside triphosphates were used for mixed tailing reactions catalyzed by TENT4A and TENT4B^2^. However, quantifying the in vivo mixed tailing rate remains a challenge. Existing sequencing methods such as TAIL-seq underestimate mixed tailing frequency^1^ and must account for multiple trimming enzymes involved in poly(A) tail modifications, which substantial affect the tail sequences. Nevertheless, our data suggest that mixed tails in the context of in vitro transcribed mRNAs may help stabilize the RNA and increase the duration of gene expression for applications in vaccination and gene therapy. For example, if a synthetic mixed tail of 100 nt contains ten intermittent guanosines, uridines or cytidines, that will be equivalent to a pure poly(A) of 150, 170 or 200 nt, respectively. This equates to up to a twofold increase in the time required to complete the shortening of the poly(A) tail.

In the case of CAF1, a non-A corresponds to around 8 adenosines, but for CCR4, a single pyrimidine is equivalent to 18 adenosines. This striking difference demonstrates that CCR4 is much more sensitive to non-A than CAF1 and uncovers the distinct nucleotide specificity of these two deadenylases. In human cells, CCR4 and CAF1 play largely redundant roles in regulating the poly(A) tail, but it is plausible that their relative contribution may vary depending on the transcript and their associated factors^17,19,20,35–37^. Our mathematical framework can be used to dissect the exact impact of individual regulatory factors such as PABP, GW182, TOB and PAIP proteins by reconstituting a biochemical deadenylation system with these factors and longer RNA molecules. Extending this towards the modeling of this dynamic process within cells when, for example, combined with an inducible expression system and poly(A) tail length measurement assay will be the focus for future work. Of note, although other deadenylases such as PAN2/PAN3 and PARN do not play a major role in mRNA deadenylation, they may participate in shortening mixed tails and are worth further investigation^18^.

While the physiological context of mixed tailing remains poorly understood, it is worth emphasizing that mixed tailing is critical for some viruses. In unbiased clustered regularly interspaced short palindromic repeats knockout screens, TENT4 and its co-factor ZCCHC14 were identified as critical pro-viral factors for the replication of hepatitis A virus^38^ and hepatitis B virus^9^. TENT4 and ZCCHC14 also mediate the mixed tailing of HCMV RNA2.7^10^. This unexpected convergent evolution across three unrelated viral families (*Picornaviridae*, *Hepadnaviridae* and *Herpesviridae*) highlights the regulatory potency and importance of mixed tailing. Mixed tailing may also be important in animal development. *Caenorhabditis* *elegans* homolog gld-4 is highly expressed in germ cells and required for meiotic progression^39^. It was also reported recently that mixed tailing increases following fertilization and decreases later in human embryo development^40^. All in all, our current study provides the means to interpret the impact of mixed tailing on mRNA deadenylation from a quantitative perspective.

## Methods

### Protein purification

Detailed protocols for purification and reconstitution of the full human CCR4–NOT complex and its variants are described in our previous paper^20^. Briefly, the full-length CNOT1, CNOT2, CNOT3 and CNOT9/CAF40 proteins were recombinantly co-produced using baculovirus-infected Sf21 insect cells (Thermo Fisher Scientific, catalog no. 11497013) and the heterotetrameric subcomplex was purified using affinity chromatography. The heterodimeric subcomplexes of CNOT10:CNOT11 and CNOT6/CCR4a:CNOT7/CAF1 were recombinantly produced and purified from BL21 (DE3) Star *Escherichia* *coli* cells (Thermo Fisher Scientific, catalog no. C601003) using chromatographic separation. The eight-subunit full complex was assembled from three purified subcomplexes and separated by size exclusion chromatography.

### In vitro deadenylation assay

In vitro deadenylation assays were done as described previously with minor modifications. Deadenylation reactions were carried out at 37 °C in a buffer containing 20 mM PIPES pH 7.0, 40 mM NaCl, 10 mM KCl and 2 mM Mg(OAc)_2_. A purified human CCR4–NOT complex (25 nM) was mixed with a synthetic 5′-fluorescein-labeled RNA substrate (50 nM), and the reaction was stopped at the corresponding time point by adding 3× reaction volumes of RNA loading dye (95% (v/v) deionized formamide, 17.5 mM EDTA pH 8 and 0.01% (w/v) bromophenol blue). The reaction products were resolved on a denaturing Tris/borate/ethylenediaminetetraacetic acid–urea polyacrylamide gel, which was subsequently imaged using an Amersham Typhoon Biomolecular Imager (Cytiva).

### RNA substrate preparation

The RNAs are labeled with 6-carboxyfluorescein (fluorescein derivative) at their 5′ ends. Sequences and names are listed in Supplementary Table 1. The synthetic RNAs were purchased from biomers.net GmbH.

### Data pre-processing and visualization

RNA intensity levels of the in vitro deadenylation assays were quantified by Multi Gauge V3.0 (Fujifilm). Horizontal alignment with the marker lane (that is, A20, A1, A0 and UCU) enabled the identification of the poly(A) tail length of each RNA species. The second-order difference (that is, discrete analog of the second derivative) was computed over the horizontal sum of pixel intensity values to identify the vertical pixel positions that separate each RNA species. These positions were then finely adjusted by manual inspection. The maximum value within these vertical positions was computed, and unity-based normalization was applied across the in vitro deadenylation assay. For data visualization, we employed the heatmap with the viridis color scheme. Column-specific unity-based normalization was applied to highlight the most abundant RNA species for that particular deadenylation experiment.

### Mathematical model of deadenylation

We make two mathematical assumptions to model the deadenylation process at single-nucleotide resolution. First, we assume that deadenylation for each nucleotide follows the first-order Markov property, where the amount of RNA for any given state only depends on its previous state. In other words, this model is a first-order stochastic process where its state space is defined by the poly(A) tail length. For example, the amount of A18 RNAs (that is, RNA with poly(A) tail of length 18) will depend on the amount of A19 RNAs but will be independent of the amount of A20 RNAs. Second, we assume that the deadenylation rate at each nucleotide is time independent. That is, the deadenylation rate of A18 RNAs is fixed across reaction time points (for example, 12 min and 48 min). These two assumptions lead to the following mathematical model:

$$\frac{{\mathrm{d}}{x}_{i}}{{{\mathrm{d}}t}}={\lambda }_{i+1}{x}_{i+1}-{\lambda }_{i}{x}_{i},$$

where ${{{x}}}_{{{i}}}$ is the amount of RNA with poly(A) tail of length *i*, and ${\lambda }_{i}$ is the deadenylation rate (or kinetics) of RNA with poly(A) tail of length *i*.

The mathematical model of deadenylation defined above is a nonlinear system of ordinary differential equations. The cost function for parameter optimization is the residual or the difference between the observed and predicted values. Specifically, the observed values are the unity-based normalized intensity values from the experiment as mentioned in the above section. The predicted values are generated via computer simulation by the deSolve R package^41^. The parameters (that is, deadenylation kinetics) were estimated by the LM algorithm^29^ as implemented in the minpack.lm R package^42^. The damping parameter is chosen on the basis of the LM implementation from the MINPACK FORTRAN library, which consists of modules for solving systems of nonlinear equations. The robustness of parameter estimation was confirmed by fitting the model with a subset of the dataset aside for cross-validation. The key pre-processing step in the context of parameter estimation is the unity-based normalization step that is applied across the in vitro deadenylation assay. The standard errors are computed on the basis of the Hessian at the parameter estimates (that is, estimation of deadenylation kinetics) and represent the range in which the true parameter values reside. That is, non-overlapping error bars suggest substantial change in deadenylation kinetics.

A truncated model was used in the case of the CCR4:CAF1^D40A^ experiments for reliable parameter estimation. Specifically, the deadenylation model was truncated at position 16 from the 3′ end of the poly(A) tail. In addition, marginal RNA levels beyond position 16 were aggregated and considered additional RNA molecules of position 16 to avoid under-estimation at or near position 16. Of note, the choice of exact truncation position did not substantial affect parameter estimation, given sufficient RNA levels up to that position.

### Quantifying the stalling effect in terms of the number of additional adenosines

The multiplicative inverse of single-nucleotide deadenylation kinetics (for example, nt min^−1^) is equivalent to the reaction time for a single deadenylation event. Therefore, comparing this reaction time between adenosine and any other non-A leads to quantitative estimation of the intrinsic kinetic property of the deadenylase of interest. That is, the stalling effect in terms of the number of additional adenosines. However, measuring the effect of intermittent non-A incorporation, also known as mixed tailing, requires handling additional biochemical features of our experimental design. First, the single removal of adenosine is not constant but slows down for RNAs with short poly(A) tails. Inferring the kinetics of ‘no stalling’ is needed for reaction time comparison. Second, the stalling effect begins at the antepenultimate and penultimate positions of non-A residue. In other words, the deadenylase is stalled at three positions from single non-A incorporation.

To address these challenges, we assume that the change in reaction time is independent for each of the three positions (that is, −2, −1 and 0). Specifically, we define the stalling effect size ${{\zeta }}$ as

$${\zeta }=\frac{z(-2)}{{z}_{{{\varnothing }}}(-2)}+\frac{z(-1)}{{z}_{{{\varnothing }}}(-1)}+\frac{z(-0)}{{z}_{{{\varnothing }}}(-0)}-2,$$

where $z(i)$ represents the reaction time for removing the nucleotide at position $i$ relative to the non-adenosine residue and ${z}_{\varnothing }(i)$ represents the reaction time of ‘no stalling’ at position $i$. For example, *z*(−2), *z*(−1) and z(−0) is the reaction time required to remove the nucleotide at positions −2, −1 and 0, respectively, relative to the non-A residue. In contrast, ${z}_{\varnothing }$ is the hypothetical reaction time if the deadenylase exhibits no stalling but only slows down as in the A20 control experiments.

To infer the hypothetical kinetics of ‘no stalling’, we consider that the gradual slowdown of deadenylation in the mixed tail (for example, A20G) experiments is proportional to that in the pure poly(A) tail (that is, A20) experiments, thus

$${{z}}\left({{i}}\right)-{{z}}\left(\,\,{{j}}\right)\propto {{{z}}}_{{\rm{A}}}\left(i\right)-{z}_{A}(\,\,j)$$

for position *i* and *j* where *i* < *j*. In other words, the average rate of slowdown is invariable across independent deadenylation experiments. Consequently, the kinetics of ‘no stalling’ ${z}_{\varnothing }$ is then

$${z}_{{{\varnothing }}}\left(i\right)=z\left(-3\right)-\epsilon (i),$$

$$\epsilon (i)=b \cdot ({z}_{\rm{A}}\left(-3\right)-{z}_{\rm{A}}\left(i\right)),$$

$${{b}}=\frac{z\left(-3\right)-z(+2)}{{z}_{\rm{A}}\left(-3\right)-{z}_{\rm{A}}(+2)},$$

where $\epsilon (i)$ represents the gradual slowdown at position ${{i}}$, *b* represents the scaling constant between independent deadenylation experiments and ${z}_{\rm{A}}\left(i\right)$ is the reaction time for the A20 control experiment at relative position ${{i}}$. The exact relative positions (that is, −3 and +2) used to infer $\epsilon (i)$ and *b* did not affect the stalling effect size ${{\zeta }}$, given that the standard error of the kinetics estimation was relatively low at those positions. Note that this formulation of ‘no stalling’ is also the basis for the constant −2 in the stalling effect size ${{\zeta }}$ equation presented above.

### Reporting summary

Further information on research design is available in the Nature Portfolio Reporting Summary linked to this article.

## Online content

Any methods, additional references, Nature Portfolio reporting summaries, source data, extended data, supplementary information, acknowledgements, peer review information; details of author contributions and competing interests; and statements of data and code availability are available at 10.1038/s41594-023-01187-1.

### Supplementary information

Reporting Summary

Supplementary Table 1. List of RNA substrates.

### Source data

Source Data Fig. 2 Unprocessed gels.

Source Data Extended Data Fig. 2 Unprocessed gels.

Source Data Extended Data Fig. 3 Unprocessed gels.

Source Data Extended Data Fig. 4 Unprocessed gels.

## Data Availability

All data are available from the corresponding authors upon reasonable request. Source data are provided with this paper.

## Code availability

Computational tools for estimating the kinetics of a single deadenylation event are available at https://github.com/2yngsklab/deadenylation-kinetics.
