# Supplementary figures and images for: Deadenylation kinetics of mixed poly(A) tails at single-nucleotide resolution
Source: Nat Struct Mol Biol. 2024 Feb 19;31(5):826–34. doi: 10.1038/s41594-023-01187-1 (PMC11102861; doi:10.1038/s41594-023-01187-1)

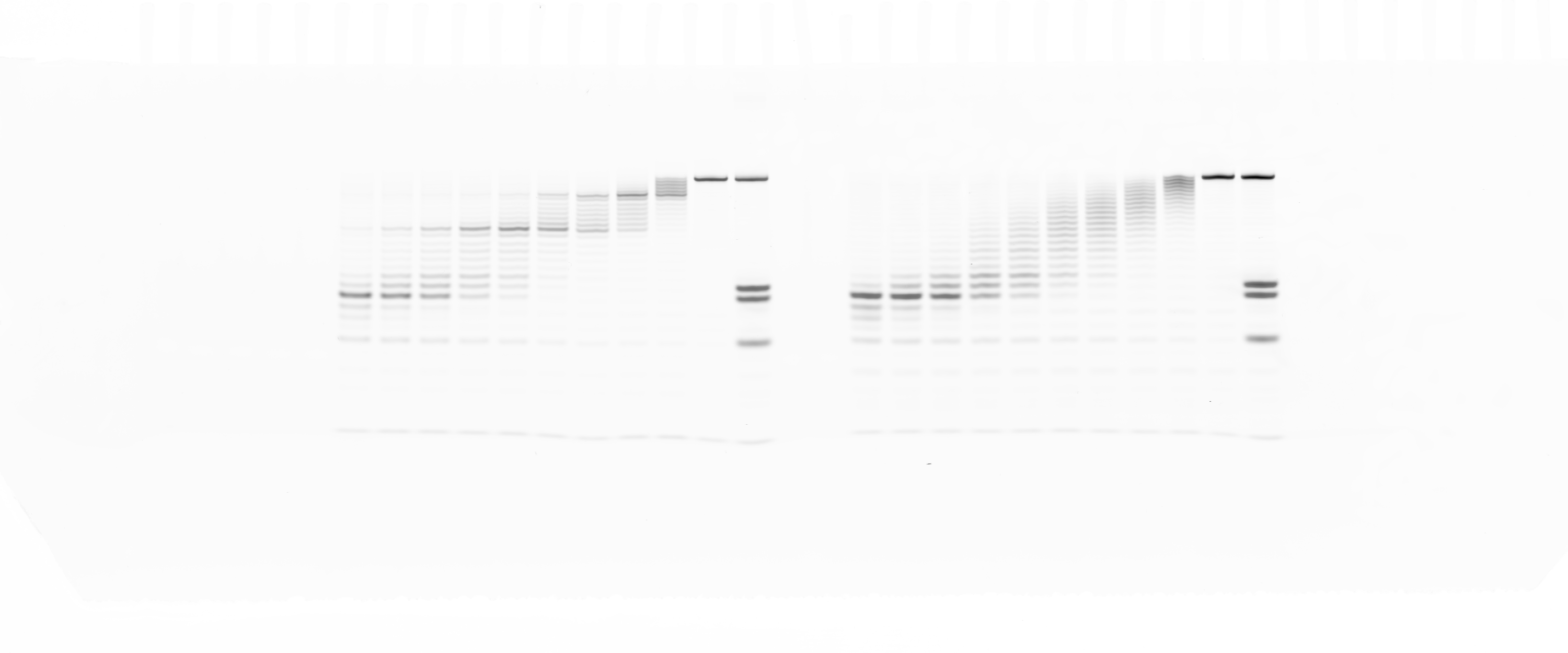

Supplement: Supplementary file 3 — Unprocessed gels. [file 41594_2023_1187_MOESM3_ESM.tif]

SD\_SFig2B

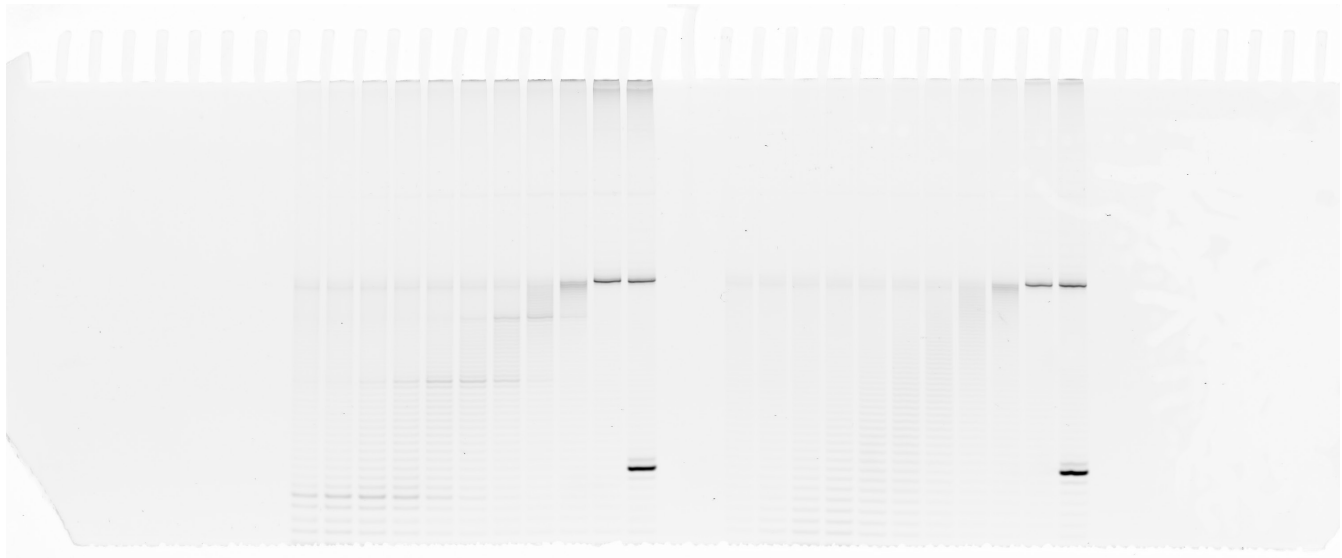

SD\_SFig2D

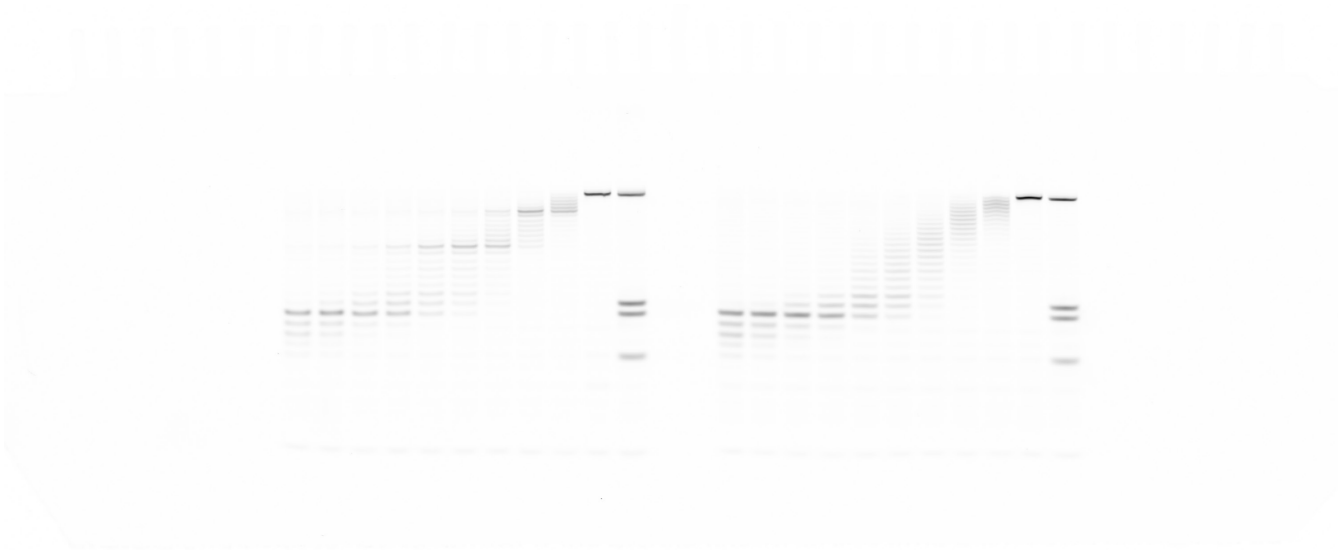

Supplement: Supplementary file 4 — Unprocessed gels. [file 41594_2023_1187_MOESM4_ESM.pdf]

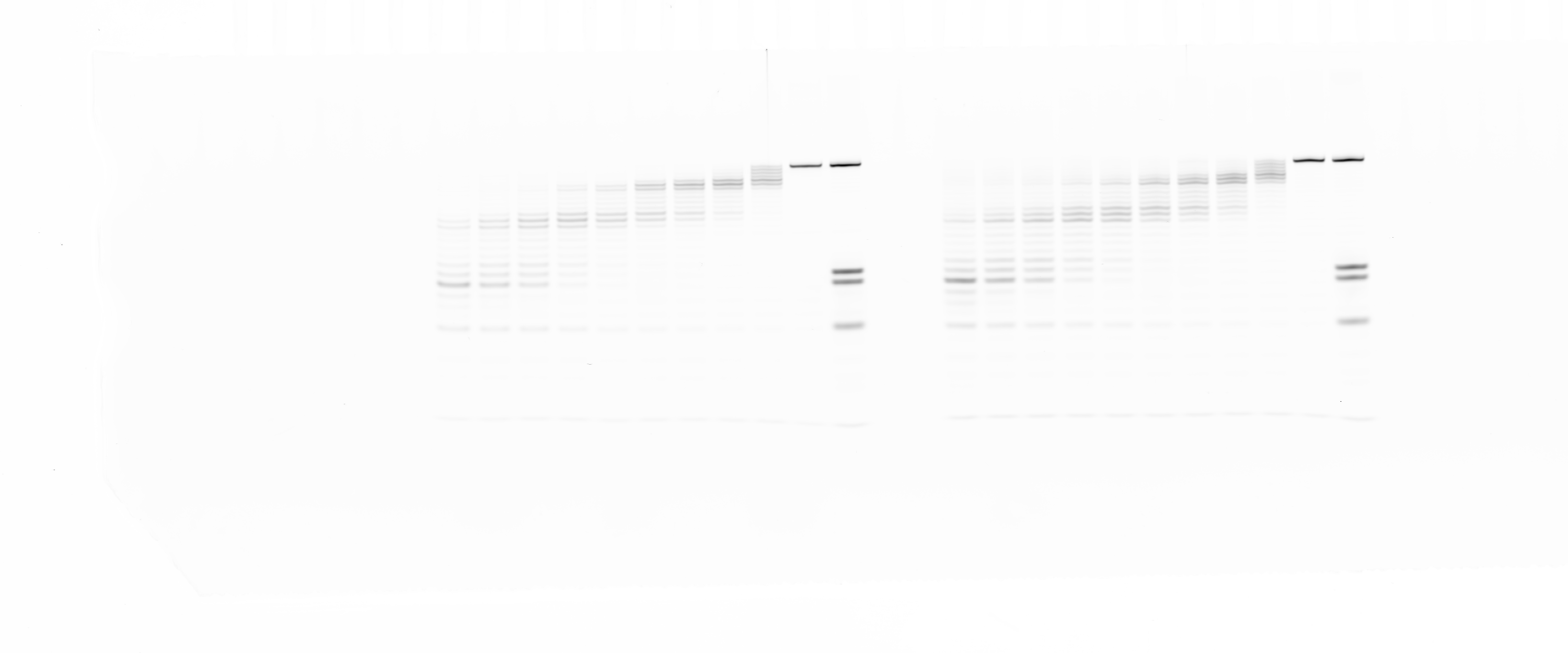

Supplement: Supplementary file 5 — Unprocessed gels. [file 41594_2023_1187_MOESM5_ESM.tif]

SD\_SFig4A

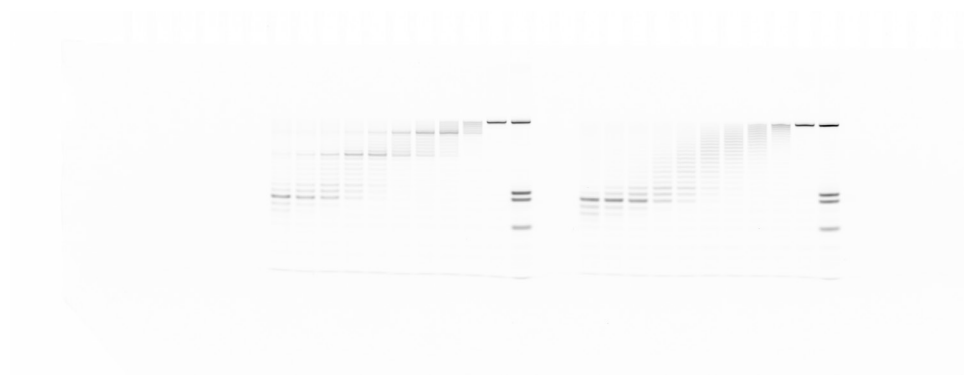

SD\_SFig4B

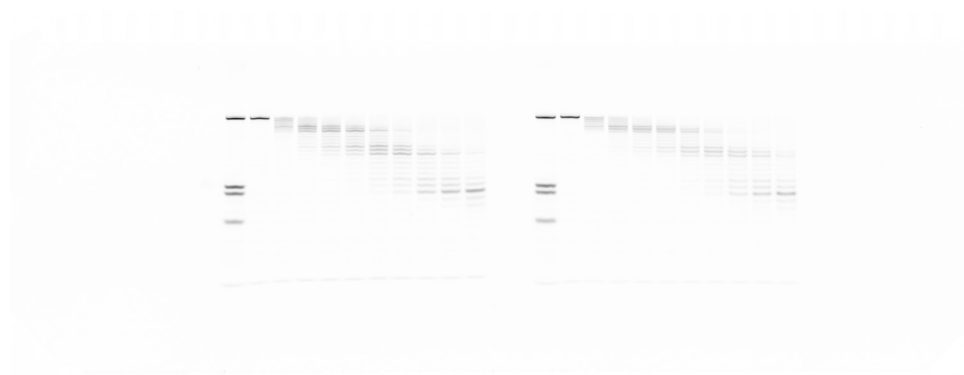

SD\_SFig4C

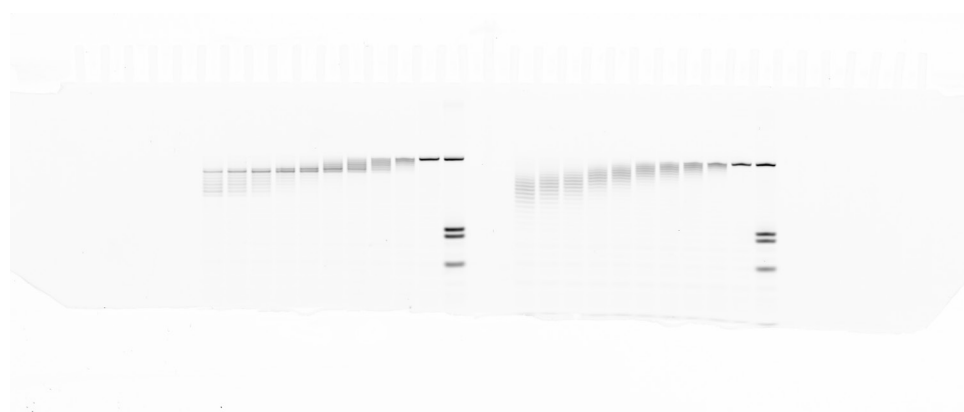

SD\_SFig4D

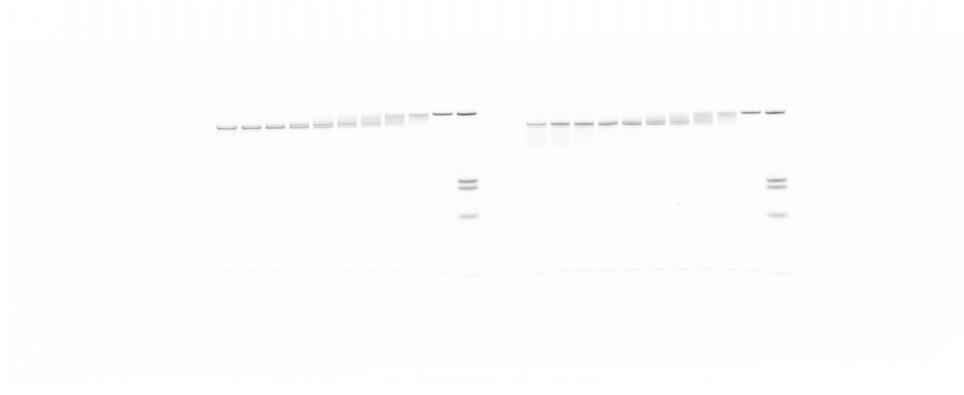

Supplement: Supplementary file 6 — Unprocessed gels. [file 41594_2023_1187_MOESM6_ESM.pdf]
